# Wavelet density estimation for mixing and size-biased data

**DOI:** 10.1186/s13660-018-1784-x

**Published:** 2018-07-25

**Authors:** Junke Kou, Huijun Guo

**Affiliations:** School of Mathematics and Computational Science, Guilin University of Electronic Technology, Guilin, P.R. China

**Keywords:** Density estimation, Strong mixing, Size-biased, Wavelets

## Abstract

This paper considers wavelet estimation for a multivariate density function based on mixing and size-biased data. We provide upper bounds for the mean integrated squared error (MISE) of wavelet estimators. It turns out that our results reduce to the corresponding theorem of Shirazi and Doosti (Stat. Methodol. 27:12–19, 2015), when the random sample is independent.

## Introduction

Let $\{Y_{i}, i\in\mathbb{Z}\}$ be a strictly stationary random process defined on a probability space $(\Omega, \mathcal{F},P)$ with the common density function

1 $$ g(y)=\frac{\omega(y)f(y)}{\mu},\quad y\in\mathbb{R}^{d}, $$

where *ω* denotes a known positive function, *f* stands for an unknown density function of the unobserved random variable *X* and $\mu =E \omega(X)=\int_{\mathbb{R}^{d}}\omega(y)f(y)\, dy<+\infty$. We want to estimate the unknown density function *f* from a sequence of strong mixing data $Y_{1}, Y_{2}, \ldots, Y_{n}$.

When $Y_{1}, Y_{2}, \ldots, Y_{n}$ are independent and $d=1$, Ramírez and Vidakovic [13] propose a linear wavelet estimator and show it to be $L^{2}$ consistent; Chesneau [1] considers the optimal convergence rates of wavelet block thresholding estimator; Shirazi and Doosti [16] expand Ramírez and Vidakovic’s [13] work to $d\geq1$. Chesneau et al. [2] extend the independence to both positively and negatively associated cases. They show a convergence rate for mean integrated squared error (MISE). An upper bound of wavelet estimation on $L^{p}$ ($1\leq p<+\infty$) risk in negatively associated case is given by Liu and Xu [9].

This paper deals with the *d*-dimensional density estimate problem (1), when $Y_{1}, Y_{2}, \ldots, Y_{n}$ are strong mixing. We give upper bounds for the mean integrated squared error (MISE) of wavelet estimators. It turns out that our linear result reduces to Shirazi and Doosti’s [16] theorem, when the random sample is independent.

### Wavelets and Besov spaces

As a central notion in wavelet analysis, Multiresolution Analysis (MRA, Meyer [11]) plays an important role in constructing a wavelet basis, which means a sequence of closed subspaces $\{V_{j}\}_{j\in \mathbb{Z}}$ of the square integrable function space $L^{2}(\mathbb {R}^{d})$ satisfying the following properties:

(i) $V_{j}\subseteq V_{j+1}$, $j\in\mathbb{Z}$. Here and after, $\mathbb {Z}$ denotes the integer set and $\mathbb{N}:=\{n\in\mathbb{Z}, n\geq0\}$;
(ii) $\overline{\bigcup_{j\in\mathbb{Z}} V_{j}}=L^{2}(\mathbb {R}^{d})$. This means the space $\bigcup_{j\in\mathbb{Z}} V_{j}$ being dense in $L^{2}(\mathbb{R}^{d})$;
(iii) $f(2\cdot)\in V_{j+1}$ if and only if $f(\cdot)\in V_{j}$ for each $j\in\mathbb{Z}$;
(iv) There exists a scaling function $\varphi\in L^{2}(\mathbb{R}^{d})$ such that $\{\varphi(\cdot-k),k\in\mathbb{Z}^{d}\}$ forms an orthonormal basis of $V_{0}=\overline{\operatorname{span}}\{\varphi(\cdot-k)\}$.

When $d=1$, there is a simple way to define an orthonormal wavelet basis. Examples include the Daubechies wavelets with compact supports. For $d\geq2$, the tensor product method gives an MRA $\{V_{j}\}$ of $L^{2}(\mathbb{R}^{d})$ from one-dimensional MRA. In fact, with a scaling function *φ* of tensor products, we find $M=2^{d}-1$ wavelet functions $\psi^{\ell}$ ($\ell=1,2,\ldots,M$) such that, for each $f\in L^{2}(\mathbb{R}^{d})$, the following decomposition

$$f=\sum_{k\in\mathbb{Z}^{d}}\alpha_{j_{0}, k} \varphi_{j_{0}, k}+\sum_{j=j_{0}}^{\infty}\sum _{\ell=1}^{M}\sum_{k\in\mathbb{Z}^{d}} \beta _{j,k}^{\ell}\psi_{j, k}^{\ell} $$

holds in $L^{2}(\mathbb{R}^{d})$ sense, where $\alpha_{j_{0},k}=\langle f,\varphi_{j_{0},k}\rangle$, $\beta_{j,k}^{\ell}=\langle f,\psi _{j,k}^{\ell}\rangle$ and

$$\varphi_{j_{0},k}(y)=2^{\frac{j_{0}d}{2}}\varphi\bigl(2^{j_{0}}y-k \bigr),\qquad \psi ^{\ell}_{j,k}(y)=2^{\frac{jd}{2}} \psi^{\ell}\bigl(2^{j}y-k\bigr). $$

Let $P_{j}$ be the orthogonal projection operator from $L^{2}(\mathbb {R}^{d})$ onto the space $V_{j}$ with the orthonormal basis $\{\varphi _{j,k}(\cdot)=2^{jd/2}\varphi(2^{j}\cdot-k),k\in\mathbb{Z}^{d}\}$. Then, for $f\in L^{2}(\mathbb{R}^{d})$,

2 $$ P_{j}f=\sum_{k\in\mathbb{Z}^{d}} \alpha_{j,k}\varphi_{j,k}. $$

A wavelet basis can be used to characterize Besov spaces. The next lemma provides equivalent definitions for those spaces, for which we need one more notation: a scaling function *φ* is called *m*-regular if $\varphi\in C^{m}(\mathbb{R}^{d})$ and $|D^{\alpha }\varphi(y)|\leq c(1+|y|^{2})^{-\ell}$ for each $\ell\in\mathbb{Z}$ and each multi-index $\alpha\in\mathbb{N}^{d}$ with $|\alpha|\le m$.

#### Lemma 1.1

(Meyer [11])

*Let* *φ* *be* *m*-*regular*, $\psi^{\ell} $ ($\ell=1, 2, \ldots, M$, $M=2^{d}-1 $) *be the corresponding wavelets and* $f\in L^{p}(\mathbb{R}^{d})$. *If* $\alpha_{j,k}=\langle f,\varphi_{j,k} \rangle$, $\beta_{j,k}^{\ell}=\langle f,\psi_{j,k}^{\ell } \rangle$, $p,q\in[1,\infty]$, *and* $0< s< m$, *then the following assertions are equivalent*:

1. $f\in B^{s}_{p,q}(\mathbb{R}^{d})$;
2. $\{2^{js}\|P_{j+1}f-P_{j}f\|_{p}\}\in l_{q}$;
3. $\{2^{j(s-\frac{d}{p}+\frac{d}{2})}\|\beta_{j}\|_{p}\}\in l_{q}$.

*The Besov norm of* *f* *can be defined by*

$$\Vert f \Vert _{B^{s}_{p,q}}:= \bigl\Vert (\alpha_{j_{0}}) \bigr\Vert _{p}+ \bigl\Vert \bigl(2^{j (s-\frac{d}{p}+\frac{d}{2})} \Vert \beta_{j} \Vert _{p}\bigr)_{j\geq j_{0}} \bigr\Vert _{q} \quad \textit{with } \Vert \beta_{j} \Vert _{p}^{p}=\sum_{\ell =1}^{M} \sum_{k\in\mathbb{Z}^{d}} \bigl\vert \beta^{\ell}_{j,k} \bigr\vert ^{p}. $$

### Estimators and result

In this paper, we require $\operatorname{supp} Y_{i} \subseteq[0,1]^{d}$ in model (1). This is similar to Chesneau [1], Chesneau et al. [2], Liu and Xu [9]. Now we give the definition of strong mixing.

#### Definition 1.1

(Rosenblatt [15])

A strictly stationary sequence of random vectors $\{Y_{i}\}_{i\in\mathbb{Z}}$ is said to be strong mixing if

$$\lim_{k\rightarrow\infty}\alpha(k)=\lim_{k\rightarrow\infty}\sup\bigl\{ \bigl\vert \mathbb{P} (A\cap B)-\mathbb{P}(A) \mathbb{P} (B) \bigr\vert : A\in \digamma ^{0}_{-\infty}, B\in\digamma^{\infty}_{k} \bigr\} =0, $$

where $\digamma^{0}_{-\infty} $ denotes the *σ* field generated by $\{Y_{i}\}_{i \leq0}$ and $\digamma^{\infty}_{k} $ does by $\{ Y_{i}\}_{i \geq k}$.

Obviously, the independent and identically distributed (*i.i.d.*) data are strong mixing since $\mathbb{P} (A\cap B)=\mathbb{P}(A) \mathbb{P} (B)$ and $\alpha(k)\equiv0$ in that case. Now, we provide two examples for strong mixing data.

#### Example 1

Let $X_{t}=\sum_{j\in\mathbb{Z}}a_{j}\varepsilon_{t-j}$ with

$$\{\varepsilon_{t}, t\in\mathbb{Z}\}\overset{\mathrm{i.i.d.}}{\sim} N \bigl(0, \sigma^{2}\bigr) \quad \mbox{and}\quad a_{k}= \textstyle\begin{cases} 2^{-k}, & k\geq0, \\ 0, & k< 0. \end{cases} $$

Then it can be proved by Theorem 2 and Corollary 1 of Doukhan [5] on p. 58 that $\{X_{t}, t\in\mathbb{Z}\}$ is a strong mixing sequence.

#### Example 2

Let $\{\varepsilon(t),t\in\mathbb{Z}\}\overset {\mathrm{i.i.d.}}{\sim} N_{r}(\vec{0},\Sigma)$ (*r*-dimensional normal distribution) and $\{Y(t), t\in\mathbb{Z}\}$ satisfy the auto-regression moving average equation

$$\sum_{i=0}^{p}B(i)Y(t-i)=\sum _{k=0}^{q}A(k)\varepsilon(t-k) $$

with $l\times r$ and $l\times l$ matrices $A(k)$, $B(i)$ respectively, as well as $B(0)$ being the identity matrix. If the absolute values of the zeros of the determinant $\operatorname{det} P(z):=\operatorname{det}\sum_{i=0}^{p}B(i)z^{i}$ ($z\in\mathbb{C}$) are strictly greater than 1, then $\{Y(t), t\in\mathbb{Z}\}$ is strong mixing (Mokkadem [12]).

It is well known that a Lebesgue measurable function maps *i.i.d.* data to *i.i.d.* data. When dealing with strong mixing data, it seems necessary to require the functions *ω* in (1) to be Borel measurable. A Borel measurable function *f* on $\mathbb{R}^{d}$ means $\{y\in\mathbb{R}^{d}, f(y)>c\}$ being a Borel set for each $c\in\mathbb {R}$. In that case, we can prove easily that $\{f(Y_{i})\}$ remains strong mixing and $\alpha_{f(Y)}(k)\leq\alpha_{Y}(k)$ ($k=1, 2, \ldots$) if $\{Y_{i}\}$ has the same property, see Guo [6]. This note is important for the proofs of the lemmas in the next section.

Before introducing our estimators, we formulate the following assumptions:

1. The weight function *ω* has both positive upper and lower bounds, i.e., for $y\in[0,1]^{d}$,
  $$0< c_{1}\leq\omega(y)\leq c_{2}< +\infty. $$
2. The strong mixing coefficient of $\{Y_{i}, i=1, 2, \ldots, n\}$ satisfies $\alpha(k)=O(\gamma e^{-c_{3}k})$ with $\gamma>0$, $c_{3}>0$.
3. The density $f_{(Y_{1}, Y_{k+1})}$ of $(Y_{1}, Y_{k+1})$ ($k\geq1$) and the density $f_{Y_{1}}$ of $Y_{1}$ satisfy that for $(y, y^{*})\in [0,1]^{d}\times[0,1]^{d}$,
  $$\sup_{k\geq1}\sup_{(y,y^{*})\in[0,1]^{d}\times [0,1]^{d}} \bigl\vert h_{k}\bigl(y,y^{*}\bigr) \bigr\vert \leq c_{4}, $$
  where $h_{k}(y, y^{*})=f_{(Y_{1}, Y_{k+1})}(y, y^{*})-f_{Y_{1}}(y)f_{Y_{k+1}}(y^{*})$ and $c_{4}>0$.

Assumption A1 is standard for the nonparametric density model with size-biased data, see Ramírez and Vidakovic [13], Chesneau [1], Liu and Xu [9]. Condition A3 can be viewed as a ‘Castellana–Leadbetter’ type condition in Masry [10].

We choose a *d*-dimensional scaling function

$$\varphi(y)=\varphi(y_{1},\ldots,y_{d}):=D_{2N}(y_{1}) \cdot\cdots\cdot D_{2N}(y_{d}) $$

with $D_{2N}(\cdot)$ being the one-dimensional Daubechies scaling function. Then *φ* is *m*-regular ($m>0$) when *N* gets large enough. Note that $D_{2N}$ has compact support $[0,2N-1]$ and the corresponding wavelet has compact support $[-N+1,N]$. Then, for $f\in L^{2}(\mathbb{R}^{d})$ with $\operatorname{supp} f\subseteq[0,1]^{d}$ and $M=2^{d}-1$,

$$ f(y)=\sum_{k\in\Lambda_{j_{0}}}\alpha_{j_{0},k} \varphi_{j_{0},k}(y)+\sum_{j=j_{0}}^{\infty} \sum_{\ell=1}^{M}\sum _{k\in\Lambda_{j}}\beta _{j,k}^{\ell} \psi_{j,k}^{\ell}(y), $$

where $\Lambda_{j_{0}}=\{1-2N, 2-2N, \ldots, 2^{j_{0}}\}^{d}$, $\Lambda _{j}=\{-N, -N+1, \ldots, 2^{j}+N-1\}^{d}$ and

$$ \alpha_{j_{0},k}= \int_{[0,1]^{d}}f(y)\varphi_{j_{0},k}(y)\,dy,\qquad \beta _{j,k}^{\ell}= \int_{[0,1]^{d}}f(y)\psi_{j,k}^{\ell}(y)\,dy. $$

We introduce

3 $$\begin{aligned}& \widehat{\mu}_{n}= \Biggl[\frac{1}{n}\sum _{i=1}^{n}\frac{1}{ \omega (Y_{i})} \Biggr]^{-1}, \end{aligned}$$

4 $$\begin{aligned}& \widehat{\alpha}_{j_{0},k}=\frac{\widehat{\mu}_{n}}{n}\sum _{i=1}^{n}\frac{\varphi_{j_{0},k}(Y_{i})}{\omega(Y_{i})}, \end{aligned}$$

and

5 $$ \widehat{\beta}_{j,k}^{\ell}=\frac{\widehat{\mu}_{n}}{n} \sum_{i=1}^{n}\frac{\psi_{j,k}^{\ell}(Y_{i})}{\omega(Y_{i})}. $$

Now, we define our linear wavelet estimator

6 $$ \widehat{f}^{\mathrm{lin}}_{n}(y)=\sum _{k\in\Lambda_{j_{0}}}\widehat{\alpha }_{j_{0},k}\varphi_{j_{0},k}(y) $$

and the nonlinear wavelet estimator

7 $$ \widehat{f}^{\mathrm{non}}_{n}(y)=\widehat{f}^{\mathrm{lin}}_{n}(y)+ \sum_{j=j_{0}}^{j_{1}}\sum _{\ell=1}^{M}\sum_{k\in\Lambda_{j}} \widehat{\beta }_{j,k}^{\ell}I_{\{|\widehat{\beta}_{j,k}^{\ell}|\geq\kappa t_{n}\}}\psi _{j,k}^{\ell}(y) $$

with $t_{n}:=\sqrt{\frac{\ln n}{n}}$. The positive integers $j_{0}$ and $j_{1}$ are specified in the theorem, while the constant *κ* will be chosen in the proof of the theorem.

The following notations are needed to state our theorem: For $H>0$,

$$B^{s}_{p,q}(H):=\bigl\{ f\in B^{s}_{p,q} \bigl(\mathbb{R}^{d}\bigr), \|f\| _{B^{s}_{p,q}}\leq H\bigr\} $$

and $x_{+}:=\max\{x,0\}$. In addition, $A\lesssim B$ denotes $A\leq cB$ for some constant $c>0$; $A\gtrsim B$ means $B\lesssim A$; $A\sim B$ stands for both $A\lesssim B$ and $B\lesssim A$.

#### Main theorem

*Consider the problem defined by* (1) *under assumptions* A1*–*A3. *Let* $f\in B^{s}_{p,q}(H)$ ($p,q\in[1,\infty)$, $s>\frac {d}{p}$) *and* $\operatorname{supp} f\subseteq[0,1]^{d}$. *Then the linear wavelet estimator* $\widehat{f}^{\mathrm{lin}}_{n}$ *defined in* (6) *with* $2^{j_{0}}\sim n^{\frac{1}{2s'+d}}$ *and* $s'=s-d(\frac{1}{p}-\frac {1}{2})_{+}$ *satisfies*

8a $$ E \int_{[0,1]^{d}} \bigl\vert \widehat{f}^{\mathrm{lin}}_{n}(y)-f(y) \bigr\vert ^{2}\,dy\lesssim n^{-\frac{2s'}{2s'+d}}; $$

*the nonlinear estimator in* (7) *with* $2^{j_{0}}\sim n^{\frac{1}{2m+d}}$ ($m>s$), $2^{j_{1}}\sim(\frac{n}{(\ln n)^{3}})^{\frac{1}{d}}$ *satisfies*

8b $$\begin{aligned} E \int_{[0,1]^{d}} \bigl\vert \widehat{f}^{\mathrm{non}}_{n}(y)-f(y) \bigr\vert ^{2}\,dy\lesssim (\ln n)^{3} {n}^{-\frac{2s}{2s+d}}. \end{aligned}$$

#### Remark 1

When $d=1$, ${n}^{-\frac{2s}{2s+1}}$ is the optimal convergence rate in the minimax sense for the standard nonparametric density model, see Donoho et al. [4].

#### Remark 2

When the strong mixing data $Y_{1}, Y_{2}, \ldots, Y_{n}$ reduce to independent and identically distributed (*i.i.d.*) data, the convergence rate of our linear estimator is the same as that of Theorem 3.1 in Shirazi and Doosti [16].

#### Remark 3

Compared with the linear wavelet estimator $\widehat {f}^{\mathrm{lin}}_{n}$, the nonlinear estimator $\widehat{f}^{\mathrm{non}}_{n}$ is adaptive, which means both $j_{0}$ and $j_{1}$ do not depend on *s*, *p*, and *q*. On the other hand, the convergence rate of the nonlinear estimator remains the same as that of the linear one up to $(\ln n)^{3}$, when $p\geq2$. However, it gets better for $1\leq p<2$.

## Some lemmas

In this section, we provide some lemmas for the proof of the theorem. The following simple (but important) lemma holds.

### Lemma 2.1

*For the model defined in* (1),

9a $$\begin{aligned}& E\bigl(\widehat{\mu}_{n}^{-1}\bigr)={\mu}^{-1}, \end{aligned}$$

9b $$\begin{aligned}& E \biggl[\frac{\mu\varphi_{j_{0},k}(Y_{i})}{\omega(Y_{i})} \biggr]=\alpha _{j_{0},k}, \end{aligned}$$

9c $$\begin{aligned}& E \biggl[\frac{\mu\psi^{\ell}_{j,k}(Y_{i})}{\omega(Y_{i})} \biggr]=\beta ^{\ell}_{j,k}, \end{aligned}$$

*where* $\alpha_{j_{0},k}=\int_{[0, 1]^{d}}f(y)\varphi_{j_{0},k}(y)\,dy$ *and* $\beta_{j,k}^{\ell}=\int_{[0, 1]^{d}}f(y)\psi_{j,k}^{\ell}(y)\,dy$ ($\ell=1,2,\ldots, M$).

### Proof

One includes a simple proof for completeness. By (3),

$$E\bigl(\widehat{\mu}_{n}^{-1}\bigr)= E \Biggl[ \frac{1}{n}\sum_{i=1}^{n} \frac {1}{\omega(Y_{i})} \Biggr]= E \biggl[\frac{1}{\omega(Y_{i})} \biggr]. $$

This with (1) leads to

$$E\bigl(\widehat{\mu}_{n}^{-1}\bigr)= \int_{[0, 1]^{d}}\frac{g(y)}{\omega (y)}\,dy=\frac{1}{\mu} \int_{[0, 1]^{d}}f(y)\,dy=\frac{1}{\mu}, $$

which concludes (9a). Using (1), one knows that

$$ E \biggl[\frac{\mu\varphi_{j_{0},k}(Y_{i})}{\omega(Y_{i})} \biggr]= \int_{[0, 1]^{d}}\frac{\mu\varphi_{j_{0},k}(y)}{\omega(y)}g(y)\,dy= \int_{[0, 1]^{d}}f(y)\varphi_{j_{0},k}(y)\,dy= \alpha_{j_{0},k}. $$

This completes the proof of (9b). Similar arguments show (9c). □

To estimate $E |\widehat{\alpha}_{j_{0},k}-\alpha_{j_{0},k} |^{2}$ and $E |\widehat{\beta}^{\ell}_{j,k}-\beta^{\ell}_{j,k} |^{2}$, we introduce an important inequality, which can be found in Davydov [3].

### Davydov’s inequality

Let $\{Y_{i}\}_{i\in\mathbb{Z}}$ be strong mixing with mixing coefficient $\alpha(k)$, *f* and *g* be two measurable functions. If $E|f(Y_{1})|^{p}$ and $E|g(Y_{1})|^{q}$ exist for $p, q>0$ and $\frac{1}{p}+\frac{1}{q}<1$, then there exists a constant $c>0$ such that

$$\bigl\vert \operatorname{cov} \bigl(f(Y_{1}), g(Y_{k+1}) \bigr) \bigr\vert \leq c\bigl[\alpha (k)\bigr]^{1-\frac{1}{p}-\frac{1}{q}}\bigl[E \bigl\vert f(Y_{1}) \bigr\vert ^{p} \bigr]^{\frac{1}{p}} \bigl[E \bigl\vert g(Y_{1}) \bigr\vert ^{q} \bigr]^{\frac{1}{q}}. $$

### Lemma 2.2

*Let* $f\in B^{s}_{p,q}(H)$ ($p,q\in[1,\infty)$, $s>\frac{d}{p}$) *and* $\widehat{\alpha}_{j_{0},k}$, $\widehat{\beta}^{\ell }_{j,k}$ *be defined by* (4) *and* (5). *If* A1*–*A3 *hold*, *then*

$$ E \vert \widehat{\alpha}_{j_{0},k}-\alpha_{j_{0},k} \vert ^{2}\lesssim n^{-1},\qquad E \bigl\vert \widehat{ \beta}^{\ell}_{j,k}-\beta^{\ell}_{j,k} \bigr\vert ^{2}\lesssim n^{-1}. $$

### Proof

One proves the second inequality only, the first one is similar. By the definition of $\widehat{\beta}^{\ell}_{j,k}$,

$$ \widehat{\beta}^{\ell}_{j,k}-\beta^{\ell}_{j,k} =\frac{\widehat{\mu}_{n}}{\mu} \Biggl[\frac{\mu}{n}\sum_{i=1}^{n} \frac {\psi^{\ell}_{j,k}(Y_{i})}{\omega(Y_{i})}-\beta^{\ell}_{j,k} \Biggr]+ \beta^{\ell}_{j,k}\cdot\widehat{\mu}_{n} \biggl( \frac{1}{\mu}-\frac {1}{\widehat{\mu}_{n}} \biggr) $$

and $E \vert \widehat{\beta}^{\ell}_{j,k}-\beta^{\ell}_{j,k} \vert ^{2}\lesssim E \vert \frac{\widehat{\mu}_{n}}{\mu} [\frac{\mu}{n}\sum_{i=1}^{n}\frac{\psi^{\ell}_{j,k}(Y_{i})}{\omega(Y_{i})}-\beta^{\ell }_{j,k} ] \vert ^{2} +E \vert \beta^{\ell}_{j,k}\widehat{\mu}_{n} (\frac{1}{\mu}-\frac {1}{\widehat{\mu}_{n}} ) \vert ^{2}$. Note that $B_{p,q}^{s}(\mathbb{R}^{d})\subseteq B_{\infty,\infty }^{s-\frac{d}{p}}(\mathbb{R}^{d})$ with $s>\frac{d}{p}$. Then $f\in B_{\infty,\infty}^{s-\frac{d}{p}}(\mathbb{R}^{d})$ and $\|f\|_{\infty }\lesssim1$. Moreover, $\vert \beta^{\ell}_{j,k} \vert := \vert \int _{[0,1]^{d}}f(y) \psi^{\ell}_{j,k}(y)\,dy \vert \lesssim1$ thanks to Hölder’s inequality and orthonormality of $\{\psi^{\ell}_{j,k}\}$. On the other hand, $\vert \frac{\widehat{\mu}_{n}}{\mu} \vert \lesssim1$ and $|\widehat{\mu}_{n}|\lesssim1$ because of A1. Hence,

10 $$ E \bigl\vert \widehat{\beta}^{\ell}_{j,k}- \beta^{\ell}_{j,k} \bigr\vert ^{2}\lesssim E \Biggl\vert \frac{\mu}{n}\sum_{i=1}^{n} \frac{\psi^{\ell }_{j,k}(Y_{i})}{\omega(Y_{i})}-\beta^{\ell}_{j,k} \Biggr\vert ^{2}+E \biggl\vert \frac{1}{\mu}-\frac{1}{\widehat{\mu}_{n}} \biggr\vert ^{2}. $$

It follows from Lemma 2.1 and the definition of variance that

11 $$\begin{aligned} E \bigl\vert \widehat{\beta}^{\ell}_{j,k}- \beta^{\ell}_{j,k} \bigr\vert ^{2} \lesssim& \operatorname{var} \Biggl[\frac{1}{n}\sum_{i=1}^{n} \frac{\psi ^{\ell}_{j,k}(X_{i})}{\omega(Y_{i})} \Biggr]+\operatorname{var} \Biggl[\frac {1}{n}\sum _{i=1}^{n}\frac{1}{\omega(X_{i},Y_{i})} \Biggr] \\ =&\frac{1}{n^{2}}\operatorname{var} \Biggl[\sum _{i=1}^{n}\frac{\psi^{\ell }_{j,k}(X_{i})}{\omega(Y_{i})} \Biggr]+ \frac{1}{n^{2}}\operatorname{var} \Biggl[\sum_{i=1}^{n} \frac{1}{\omega(X_{i},Y_{i})} \Biggr]. \end{aligned}$$

Note that Condition A1 implies $\operatorname{var} (\frac{1}{\omega (Y_{i})} ) \leq E (\frac{1}{\omega(Y_{i})} )^{2}\lesssim 1$ and

$$ \operatorname{var} \Biggl[\sum_{i=1}^{n} \frac{1}{\omega(Y_{i})} \Biggr]\lesssim n \operatorname{var} \biggl(\frac{1}{\omega(Y_{i})} \biggr)+ \Biggl\vert \sum_{v=2}^{n}\sum _{i=1}^{v-1}\operatorname{cov} \biggl( \frac{1}{\omega(Y_{v})}, \frac{1}{\omega(Y_{i})} \biggr) \Biggr\vert . $$

Then it suffices to show

12 $$ \Biggl\vert \sum_{v=2}^{n} \sum_{i=1}^{v-1}\operatorname{cov} \biggl( \frac{1}{\omega (Y_{v})}, \frac{1}{\omega(Y_{i})} \biggr) \Biggr\vert \lesssim n. $$

By the strict stationarity of $Y_{i}$,

$$\begin{aligned} \Biggl\vert \sum_{v=2}^{n}\sum _{i=1}^{v-1}\operatorname{cov} \biggl( \frac{1}{\omega (Y_{v})}, \frac{1}{\omega(Y_{i})} \biggr) \Biggr\vert =& \Biggl\vert \sum_{m=1}^{n}(n-m) \operatorname{cov} \biggl(\frac{1}{\omega(Y_{1})}, \frac{1}{\omega(Y_{m+1})} \biggr) \Biggr\vert \\ \leq& n\sum_{m=1}^{n} \biggl\vert \operatorname{cov} \biggl(\frac{1}{\omega(Y_{1})}, \frac{1}{\omega(Y_{m+1})} \biggr) \biggr\vert . \end{aligned}$$

On the other hand, Davydov’s inequality and A1 show that

$$ \biggl\vert \operatorname{cov} \biggl(\frac{1}{\omega(Y_{1})}, \frac{1}{\omega (Y_{m+1})} \biggr) \biggr\vert \lesssim\sqrt{\alpha(m)}\sqrt{E \biggl\vert \frac {1}{\omega(Y_{1})} \biggr\vert ^{4}}\lesssim\sqrt{\alpha(m)}. $$

These with A2 give the desired conclusion (12),

$$ \Biggl\vert \sum_{v=2}^{n}\sum _{i=1}^{v-1}\operatorname{cov} \biggl( \frac{1}{\omega (Y_{v})}, \frac{1}{\omega(Y_{i})} \biggr) \Biggr\vert \lesssim n\sum _{m=1}^{n}\sqrt{\alpha(m)}\lesssim n. $$

Now, the main work is to show

13 $$ \operatorname{var} \Biggl[\sum_{i=1}^{n} \frac{\psi^{\ell}_{j,k}(X_{i})}{\omega (Y_{i})} \Biggr]\lesssim n. $$

Clearly,

$$ \operatorname{var} \Biggl[\sum_{i=1}^{n} \frac{\psi^{\ell}_{j,k}(Y_{i})}{\omega (Y_{i})} \Biggr] \lesssim n \operatorname{var} \biggl(\frac{\psi^{\ell }_{j,k}(Y_{i})}{\omega(Y_{i})} \biggr)+ \Biggl\vert \sum_{v=2}^{n}\sum _{i=1}^{v-1}\operatorname{cov} \biggl( \frac{\psi^{\ell}_{j,k}(Y_{v})}{\omega (Y_{v})},\frac{\psi^{\ell}_{j,k}(Y_{i})}{\omega(Y_{i})} \biggr) \Biggr\vert . $$

By A1–A3 and (1), the first term of the above inequality is bounded by

$$n E \biggl(\frac{\psi^{\ell}_{j,k}(Y_{i})}{\omega(Y_{i})} \biggr)^{2}\lesssim n \int_{[0, 1]^{d}} \bigl[\psi^{\ell}_{j,k}(y) \bigr]^{2}f(y)\,dy\lesssim n. $$

It remains to show

14 $$\begin{aligned} \begin{aligned}[b] &\Biggl\vert \sum_{v=2}^{n}\sum _{i=1}^{v-1}\operatorname{cov} \biggl( \frac{\psi^{\ell }_{j,k}(Y_{v})}{\omega(Y_{v})},\frac{\psi^{\ell}_{j,k}(Y_{i})}{\omega (Y_{i})} \biggr) \Biggr\vert \\ &\quad \leq n \Biggl(\sum_{m=1}^{2^{jd}-1}+\sum _{m=2^{jd}}^{n} \Biggr) \biggl\vert \operatorname{cov} \biggl[\frac{\psi^{\ell}_{j,k}(Y_{1})}{\omega(Y_{1})},\frac {\psi^{\ell}_{j,k}(Y_{m+1})}{\omega(Y_{m+1})} \biggr] \biggr\vert \lesssim n, \end{aligned} \end{aligned}$$

where the assumption $2^{jd}\leq n$ is needed.

According to A1 and A3,

$$\begin{aligned} \biggl\vert \operatorname{cov} \biggl(\frac{\psi^{\ell}_{j,k}(Y_{1})}{\omega (Y_{1})},\frac{\psi^{\ell}_{j,k}(Y_{m+1})}{\omega(Y_{m+1})} \biggr) \biggr\vert \leq& \int_{[0,1]^{d}\times[0,1]^{d}} \biggl\vert \frac{\psi^{\ell }_{j,k}(y)}{\omega(y)}\cdot \frac{\psi^{\ell}_{j,k}(y^{*})}{\omega (y^{*})} \biggr\vert \bigl\vert h_{m} \bigl(y,y^{*}\bigr) \bigr\vert \, dy\, dy^{*} \\ \lesssim& \biggl( \int_{[0,1]^{d}} \bigl\vert \psi^{\ell}_{j,k}(y) \bigr\vert \,dy \biggr)^{2}\lesssim2^{-jd}. \end{aligned}$$

Hence,

15 $$ \sum_{m=1}^{2^{jd}-1} \biggl\vert \operatorname{cov} \biggl(\frac{\psi^{\ell }_{j,k}(Y_{1})}{\omega(Y_{1})},\frac{\psi^{\ell}_{j,k}(Y_{m+1})}{\omega (Y_{m+1})} \biggr) \biggr\vert \lesssim\sum_{m=1}^{2^{jd}-1}2^{-jd} \lesssim1. $$

On the other hand, Davydov’s inequality and A1–A3 tell that

$$\begin{aligned} \biggl\vert \operatorname{cov} \biggl(\frac{\psi^{\ell}_{j,k}(Y_{1})}{\omega (Y_{1})},\frac{\psi^{\ell}_{j,k}(Y_{m+1})}{\omega(Y_{m+1})} \biggr) \biggr\vert \lesssim&\sqrt{\alpha(m)}\sqrt{E \biggl\vert \frac{\psi^{\ell }_{j,k}(Y_{1})}{\omega(Y_{1})} \biggr\vert ^{4}} \\ \lesssim&\sqrt{\alpha(m)}\sup \biggl\vert \frac{\psi^{\ell}_{j,k}(Y_{1})}{\omega (Y_{1})} \biggr\vert \sqrt{E \biggl\vert \frac{\psi^{\ell}_{j,k}(Y_{1})}{\omega (Y_{1})} \biggr\vert ^{2}} \lesssim\sqrt{\alpha(m)} 2^{\frac{jd}{2}}. \end{aligned}$$

Moreover, $\sum_{m=2^{jd}}^{n} \vert \operatorname{cov} (\frac{\psi^{\ell }_{j,k}(Y_{1})}{\omega(Y_{1})},\frac{\psi^{\ell}_{j,k}(Y_{m+1})}{\omega (Y_{m+1})} ) \vert \lesssim\sum_{m=2^{jd}}^{n}\sqrt{\alpha(m)} 2^{\frac{jd}{2}} \lesssim\sum_{m=1}^{n}\sqrt{m\alpha(m)}\leq \sum_{m=1}^{+\infty }m^{\frac{1}{2}}\gamma e^{-\frac{cm}{2}}<+\infty$. This with (15) shows (14). □

To prove the last lemma in this section, we need the following Bernstein-type inequality (Liebscher [7, 8], Rio [14]).

### Bernstein-type inequality

Let $(Y_{i})_{i\in\mathbb{Z}}$ be a strong mixing process with mixing coefficient $\alpha(k)$, $EY_{i}=0$, $|Y_{i}|\leq M<\infty$, and $D_{m}=\max_{1\leq j\leq 2m}\operatorname{var} (\sum_{i=1}^{j}Y_{i} )$. Then, for $\varepsilon >0$ and $n,m\in\mathbb{N}$ with $0< m\leq\frac{n}{2}$,

$$\mathbb{P} \Biggl( \Biggl\vert \sum_{i=1}^{n}Y_{i} \Biggr\vert \geq\varepsilon \Biggr) \leq4\cdot\exp \biggl\{ -\frac{\varepsilon^{2}}{16} \biggl(nm^{-1}D_{m}+\frac{1}{3}\varepsilon Mm \biggr)^{-1} \biggr\} +32\frac {M}{\varepsilon}n\alpha(m). $$

### Lemma 2.3

*Let* $f\in B^{s}_{p,q}(H)$ ($p,q\in[1,\infty)$, $s>\frac{d}{p}$), $\widehat{\beta}^{\ell}_{j,k}$ *be defined in* (5) *and* $t_{n}=\sqrt{\frac{\ln n}{n}}$. *If* A1*–*A3 *hold and* $2^{jd}\leq\frac {n}{(\ln n)^{3}}$, *then there exists a constant* $\kappa>1$ *such that*

$$\mathbb{P} \bigl( \bigl\vert \widehat{\beta}_{j,k}^{\ell}- \beta_{j,k}^{\ell } \bigr\vert \geq\kappa t_{n} \bigr) \lesssim n^{-4}. $$

### Proof

According to the arguments of (10), $\vert \widehat{\beta}_{j,k}^{\ell}-\beta_{j,k}^{\ell} \vert \lesssim \frac{1}{n} \vert \sum_{i=1}^{n} [\frac{1}{\omega(Y_{i})}-\frac {1}{\mu} ] \vert + \vert \frac{1}{n} \sum_{i=1}^{n}\frac{\mu\psi_{j,k}^{\ell }(Y_{i})}{\omega(Y_{i})}-\beta_{j,k}^{\ell} \vert $. Hence, it suffices to prove

16 $$ \begin{aligned} &\mathbb{P} \Biggl(\frac{1}{n} \Biggl\vert \sum_{i=1}^{n} \biggl[\frac{1}{\omega (Y_{i})}- \frac{1}{\mu} \biggr] \Biggr\vert \geq\frac{\kappa}{2}t_{n} \Biggr) \lesssim n^{-4}\quad \mbox{and} \\ &\mathbb{P} \Biggl( \Biggl\vert \frac{1}{n}\sum _{i=1}^{n} \biggl[\frac{\mu\psi _{j,k}^{\ell}(Y_{i})}{\omega(Y_{i})}- \beta_{j,k}^{\ell} \biggr] \Biggr\vert \geq \frac{\kappa}{2}t_{n} \Biggr) \lesssim n^{-4}. \end{aligned} $$

One shows the second inequality only, because the first one is similar and even simpler.

Define $\eta_{i}:=\frac{\mu\psi_{j,k}^{\ell}(Y_{i})}{\omega (Y_{i})}-\beta_{j,k}^{\ell}$. Then $E(\eta_{i})=0$ thanks to (9c), and $\eta_{1}, \ldots, \eta_{n}$ are strong mixing with the mixing coefficients $\alpha(k)\leq\gamma e^{-ck}$ because of Condition A2. By A1–A3, $\vert \frac{\mu\psi_{j,k}^{\ell}(Y_{i})}{\omega(Y_{i})} \vert \lesssim2^{\frac{jd}{2}}$ and

$$ |\eta_{i}|\leq \biggl\vert \frac{\mu\psi_{j,k}^{\ell}(Y_{i})}{\omega(Y_{i})} \biggr\vert +E \biggl\vert \frac{\mu\psi_{j,k}^{\ell}(Y_{i})}{\omega(Y_{i})} \biggr\vert \lesssim 2^{\frac{jd}{2}}. $$

According to the arguments of (13), $D_{m}=\max_{1\leq j\leq2m}\operatorname{var} (\sum_{i=1}^{j}\eta_{i} ) \lesssim m$. Then it follows from Bernstein-type inequality with $m=u\ln n$ (the constant *u* will be chosen later on) that

17 $$\begin{aligned} \mathbb{P} \Biggl(\frac{1}{n} \Biggl\vert \sum _{i=1}^{n}\eta_{i} \Biggr\vert \geq \frac{\kappa}{2}t_{n} \Biggr) =& \mathbb{P} \Biggl( \Biggl\vert \sum_{i=1}^{n}\eta_{i} \Biggr\vert \geq\frac{\kappa}{2}nt_{n} \Biggr) \\ \lesssim&\exp \biggl\{ -\frac{(\kappa n t_{n})^{2}}{64} \biggl(nm^{-1}D_{m}+ \frac{1}{6}\kappa n t_{n} 2^{\frac{jd}{2}}m \biggr)^{-1} \biggr\} \\ &{}+64 \frac{2^{\frac{jd}{2}}}{\kappa n t_{n}}n\gamma e^{-cm}. \end{aligned}$$

Clearly, $64 \frac{2^{\frac{jd}{2}}}{\kappa n t_{n}}n\gamma e^{-cm}\lesssim n e^{-cu\ln n}$ holds due to $t_{n}=\sqrt{\frac{\ln n}{n}}$, $2^{jd}\leq\frac{n}{(\ln n)^{3}}$ and $m=u\ln n$. Choose *u* such that $1-cu<-4$, then the second term of (17) is bounded by $n^{-4}$. On the other hand, the first one of (17) has the following upper bound:

$$ \exp \biggl\{ -\frac{\kappa^{2}\ln n}{64} \biggl(1+\frac{1}{6}\kappa\sqrt { \frac{\ln n}{n}} \biggl(\frac{n}{(\ln n)^{3}} \biggr)^{\frac{1}{2}}m \biggr)^{-1} \biggr\} \lesssim\exp \biggl\{ -\frac{\kappa^{2}\ln n}{64} \biggl(1+ \frac {1}{6}\kappa u \biggr)^{-1} \biggr\} $$

thanks to $D_{m}\lesssim m$, $2^{jd}\leq\frac{n}{(\ln n)^{3}}$ and $m=u\ln n$. Obviously, there exists sufficiently large $\kappa>1$ such that $\exp \{-\frac{\kappa^{2}\ln n}{64} (1+\frac{1}{6}\kappa u )^{-1} \}\lesssim n^{-4}$. Finally, the desired conclusion (16) follows. □

## Proof of the theorem

This section proves the theorem. The main idea of the proof comes from Donoho et al. [4].

### Proof of (8a)

Note that

18 $$\begin{aligned} \begin{aligned}[b] E\int_{[0,1]^{d}} \bigl\vert \widehat{f}^{\mathrm{lin}}_{n}(y)-f(y) \bigr\vert ^{2}\,dy&\leq E \int_{\mathbb{R}^{d}} \bigl\vert \widehat{f}^{\mathrm{lin}}_{n}(y)-f(y) \bigr\vert ^{2}\,dy \\ &= E \bigl\Vert \widehat{f}^{\mathrm{lin}}_{n}-P_{j_{0}}f \bigr\Vert _{2}^{2}+ \Vert P_{j_{0}}f-f \Vert _{2}^{2}. \end{aligned} \end{aligned}$$

It is easy to see that

$$E \bigl\Vert \widehat{f}^{\mathrm{lin}}_{n}-P_{j_{0}}f \bigr\Vert ^{2}_{2}=E \biggl\Vert \sum _{k\in\Lambda_{j_{0}}}(\widehat{\alpha}_{j_{0},k}-\alpha _{j_{0},k})\varphi_{j_{0},k} \biggr\Vert ^{2}_{2} =\sum_{k\in\Lambda_{j_{0}}} E \vert \widehat{\alpha}_{j_{0},k}- \alpha_{j_{0},k} \vert ^{2}. $$

According to Lemma 2.2, $|\Lambda_{j_{0}}|\sim2^{j_{0}d}$ and $2^{j_{0}}\sim n^{\frac{1}{2s'+d}}$,

19 $$ E \bigl\Vert \widehat{f}^{\mathrm{lin}}_{n}-P_{j_{0}}f \bigr\Vert _{2}^{2}\lesssim\frac {2^{j_{0}d}}{n}\sim n^{-\frac{2s'}{2s'+d}}. $$

When $p\geq2$, $s'=s$. By Hölder’s inequality, $f\in B_{p,q}^{s}(H)$, and Lemma 1.1,

20 $$ \|P_{j_{0}}f-f\|_{2}^{2}\lesssim \|P_{j_{0}}f-f\|_{p}^{2}\lesssim 2^{-2j_{0}s}\sim n^{-\frac{2s}{2s+d}}. $$

When $1\leq p<2$ and $s>\frac{d}{p}$, $B_{p,q}^{s}(\mathbb {R}^{d})\subseteq B_{2,\infty}^{s'}(\mathbb{R}^{d})$. Then it follows from Lemma 1.1 and $2^{j_{0}}\sim n^{\frac{1}{2s'+d}}$ that

21 $$ \|P_{j_{0}}f-f\|_{2}^{2}\lesssim\sum _{j=j_{0}}^{\infty }2^{-2js'} \lesssim2^{-2j_{0}s'}\sim n^{-\frac{2s'}{2s'+d}}. $$

This with (20) shows in both cases

22 $$ \|P_{j_{0}}f-f\|_{2}^{2}\lesssim n^{-\frac{2s'}{2s'+d}}. $$

By (18), (19), and (22),

$$ E \int_{[0,1]^{d}} \bigl\vert \widehat{f}^{\mathrm{lin}}_{n}(y)-f(y) \bigr\vert ^{2}\,dy\lesssim n^{-\frac{2s'}{2s'+d}}. $$

 □

### Proof of (8b)

By the definitions of $\widehat{f}^{\mathrm{lin}}_{n}$ and $\widehat{f}^{\mathrm{non}}_{n}$, $\widehat{f}^{\mathrm{non}}_{n}(y)-f(y)= [\widehat {f}^{\mathrm{lin}}_{n}(y)-P_{j_{0}}f(y) ]- [f(y)-P_{j_{1}+1}f(y) ] +\sum_{j=j_{0}}^{j_{1}} \sum_{\ell=1}^{M}\sum_{k\in\Lambda j} [\widehat{\beta}_{j,k}^{\ell}I_{\{|\widehat{\beta}_{j,k}^{\ell}|\geq \kappa t_{n}\}}-\beta_{j,k}^{\ell} ]\psi_{j,k}^{\ell}(y)$. Hence,

23 $$ E \int_{[0,1]^{d}} \bigl\vert \widehat{f}^{\mathrm{non}}_{n}(y)-f(y) \bigr\vert ^{2}\,dy\lesssim T_{1}+T_{2}+Q, $$

where $T_{1}:=E \|\widehat{f}^{\mathrm{lin}}_{n}-P_{j_{0}}f \|^{2}_{2}$, $T_{2}:= \|f-P_{j_{1}+1}f \|^{2}_{2}$ and

$$Q:=E \Biggl\Vert \sum_{j=j_{0}}^{j_{1}} \sum _{\ell=1}^{M}\sum_{k\in\Lambda j} \bigl[\widehat{\beta}_{j,k}^{\ell}I_{\{|\widehat{\beta}_{j,k}^{\ell }|\geq\kappa t_{n}\}}- \beta_{j,k}^{\ell} \bigr]\psi_{j,k}^{\ell} \Biggr\Vert ^{2}_{2}. $$

According to (19) and $2^{j_{0}}\sim n^{\frac{1}{2m+d}}$ ($m>s$),

$$T_{1}=E \bigl\Vert \widehat{f}^{\mathrm{lin}}_{n}-P_{j_{0}}f \bigr\Vert _{2}^{2}\lesssim \frac{2^{j_{0}d}}{n}\sim n^{-\frac{2m}{2m+d}}< n^{-\frac{2s}{2s+d}}. $$

When $p\geq2$, the same arguments as (20) shows $T_{2}= \|f-P_{j_{1}+1}f \|^{2}_{2}\lesssim2^{-2j_{1}s}$. This with $2^{j_{1}}\sim (\frac{n}{(\ln n)^{3}} )^{\frac{1}{d}}$ leads to

24 $$ T_{2}\lesssim2^{-2j_{1}s}\sim \biggl( \frac{(\ln n)^{3}}{n} \biggr)^{\frac {2s}{d}}\leq(\ln n)^{3}n^{-\frac{2s}{2s+d}}. $$

On the other hand, $B_{p,q}^{s}(\mathbb{R}^{d})\subseteq B_{2,\infty }^{s+d/2-d/p}(\mathbb{R}^{d})$ when $1\leq p<2$ and $s>\frac{d}{p}$. Then

$$ T_{2}\lesssim2^{-2j_{1}(s+\frac{d}{2}-\frac{d}{p})}\sim \biggl(\frac {(\ln n)^{3}}{n} \biggr)^{\frac{2(s+\frac{d}{2}-\frac{d}{p})}{d}}\leq(\ln n)^{3}n^{-\frac{2s}{2s+d}}. $$

Hence,

$$T_{2}\lesssim(\ln n)^{3}n^{-\frac{2s}{2s+d}} $$

for each $1\leq p<+\infty$.

The main work for the proof of (8b) is to show

25 $$ Q=E \Biggl\Vert \sum_{j=j_{0}}^{j_{1}} \sum_{\ell=1}^{M}\sum _{k\in\Lambda j} \bigl[\widehat{\beta}_{j,k}^{\ell}I_{\{|\widehat{\beta}_{j,k}^{\ell }|\geq\kappa t_{n}\}}- \beta_{j,k}^{\ell} \bigr]\psi_{j,k}^{\ell} \Biggr\Vert ^{2}_{2}\lesssim(\ln n)^{3}{n}^{-\frac{2s}{2s+d}}. $$

Note that

26 $$ Q=\sum_{j=j_{0}}^{j_{1}}\sum _{\ell=1}^{M}\sum_{k\in\Lambda_{j}}E \bigl\vert \widehat{\beta}_{j,k}^{\ell}I_{\{|\widehat{\beta}_{j,k}^{\ell}|\geq \kappa t_{n}\}}- \beta_{j,k}^{\ell} \bigr\vert ^{2}\lesssim Q_{1}+Q_{2}+Q_{3}, $$

where

$$\begin{aligned}& Q_{1}=\sum_{j=j_{0}}^{j_{1}}\sum _{\ell=1}^{M}\sum_{k\in\Lambda _{j}}E \bigl[ \bigl\vert \widehat{\beta}_{j,k}^{\ell}- \beta_{j,k}^{\ell} \bigr\vert ^{2}I_{\{|\widehat{\beta}_{j,k}^{\ell}-\beta_{j,k}^{\ell}|>\frac {\kappa t_{n}}{2}\}} \bigr], \\& Q_{2}=\sum_{j=j_{0}}^{j_{1}}\sum _{\ell=1}^{M}\sum_{k\in\Lambda _{j}}E \bigl[ \bigl\vert \widehat{\beta}_{j,k}^{\ell}- \beta_{j,k}^{\ell} \bigr\vert ^{2}I_{\{|\beta_{j,k}^{\ell}|\geq\frac{\kappa t_{n}}{2}\}} \bigr], \\& Q_{3}=\sum_{j=j_{0}}^{j_{1}}\sum _{\ell=1}^{M}\sum_{k\in\Lambda _{j}} \bigl\vert \beta_{j,k}^{\ell} \bigr\vert ^{2}I_{\{|\beta_{j,k}^{\ell}|\leq 2\kappa t_{n}\}}. \end{aligned}$$

For $Q_{1}$, one observes that

$$E \bigl[ \bigl\vert \widehat{\beta}_{j,k}^{\ell}- \beta_{j,k}^{\ell} \bigr\vert ^{2}I_{\{|\widehat{\beta}_{j,k}^{\ell}-\beta_{j,k}^{\ell}|>\frac {\kappa t_{n}}{2}\}} \bigr]\leq \bigl[E \bigl\vert \widehat{\beta}_{j,k}^{\ell }- \beta_{j,k}^{\ell} \bigr\vert ^{4} \bigr]^{\frac{1}{2}} \biggl[\mathbb{P} \biggl(\bigl|\widehat{\beta}_{j,k}^{\ell}- \beta_{j,k}^{\ell}\bigr|>\frac{\kappa t_{n}}{2} \biggr) \biggr]^{\frac{1}{2}} $$

thanks to Hölder’s inequality. By Lemmas 2.1–2.3 and $2^{jd}\leq n$,

$$ E \bigl[ \bigl\vert \widehat{\beta}_{j,k}^{\ell}- \beta_{j,k}^{\ell} \bigr\vert ^{2}I_{\{|\widehat{\beta}_{j,k}^{\ell}-\beta_{j,k}^{\ell}|>\frac {\kappa t_{n}}{2}\}} \bigr]\lesssim \biggl[\frac{2^{jd}}{n} \biggr]^{\frac {1}{2}} \biggl[ \frac{1}{n^{4}} \biggr]^{\frac{1}{2}}\lesssim\frac {1}{n^{2}}. $$

Then $Q_{1}\lesssim\sum_{j=j_{0}}^{j_{1}}\frac{2^{jd}}{n^{2}}\lesssim \frac{2^{j_{1}d}}{n^{2}}\lesssim\frac{1}{n}\leq n^{-\frac{2s}{2s+d}}$, where one uses the choice $2^{j_{1}}\sim (\frac{n}{(\ln n)^{3}} )^{\frac{1}{d}}$. Hence,

27 $$ Q_{1}\leq n^{-\frac{2s}{2s+d}}. $$

To estimate $Q_{2}$, one defines

$$2^{j'}\sim n^{\frac{1}{2s+d}}. $$

It is easy to see that $2^{j_{0}}\sim n^{\frac{1}{2m+d}}\leq2^{j'}\sim n^{\frac{1}{2s+d}}\leq2^{j_{1}}\sim (\frac{n}{(\ln n)^{3}} )^{\frac{1}{d}}$. Furthermore, one rewrites

$$\begin{aligned} Q_{2} =& \Biggl(\sum_{j=j_{0}}^{j'}+ \sum_{j=j'+1}^{j_{1}} \Biggr) \Biggl\{ \sum _{\ell=1}^{M}\sum_{k\in\Lambda_{j}}E \bigl[ \bigl\vert \widehat{\beta }_{j,k}^{\ell}- \beta_{j,k}^{\ell} \bigr\vert ^{2}I_{\{|\beta_{j,k}^{\ell }|\geq\frac{\kappa t_{n}}{2}\}} \bigr] \Biggr\} \\ :=&Q_{21}+Q_{22}. \end{aligned}$$

By Lemma 2.2 and $2^{j'}\sim n^{\frac{1}{2s+d}}$,

28 $$\begin{aligned} Q_{21} :=&\sum_{j=j_{0}}^{j'}\sum _{\ell=1}^{M}\sum_{k\in\Lambda _{j}}E \bigl[ \bigl\vert \widehat{\beta}_{j,k}^{\ell}- \beta_{j,k}^{\ell} \bigr\vert ^{2}I_{\{|\beta_{j,k}^{\ell}|\geq\frac{\kappa t_{n}}{2}\}} \bigr] \\ \lesssim&\sum_{j=j_{0}}^{j'}\sum _{\ell=1}^{M}\sum_{k\in\Lambda _{j}} \frac{1}{n} \lesssim\sum_{j=j_{0}}^{j'} \frac{2^{jd}}{n}\lesssim\frac {2^{j'd}}{n}\sim n^{-\frac{2s}{2s+d}}. \end{aligned}$$

On the other hand, it follows from Lemma 2.2 that

$$\begin{aligned} Q_{22} :=&\sum_{j=j'+1}^{j_{1}}\sum _{\ell=1}^{M}\sum_{k\in\Lambda _{j}}E \bigl[ \bigl\vert \widehat{\beta}_{j,k}^{\ell}- \beta_{j,k}^{\ell} \bigr\vert ^{2}I_{\{|\beta_{j,k}^{\ell}|\geq\frac{\kappa t_{n}}{2}\}} \bigr] \\ \lesssim&\sum_{j=j'+1}^{j_{1}}\sum _{\ell=1}^{M}\sum_{k\in\Lambda _{j}} \frac{1}{n} I_{\{|\beta_{j,k}^{\ell}|\geq\frac{\kappa t_{n}}{2}\}}. \end{aligned}$$

When $p\geq2$,

29 $$\begin{aligned} Q_{22} \lesssim&\sum_{j=j'+1}^{j_{1}} \sum_{\ell=1}^{M}\sum _{k\in \Lambda_{j}}\frac{1}{n} I_{\{|\beta_{j,k}^{\ell}|\geq\frac{\kappa t_{n}}{2}\}}\lesssim\sum _{j=j'+1}^{j_{1}}\sum_{\ell=1}^{M} \sum_{k\in \Lambda_{j}}\frac{1}{n} \biggl( \frac{\beta_{j,k}^{\ell}}{\kappa t_{n}/2} \biggr)^{2} \\ \lesssim&\sum_{j=j'+1}^{j_{1}}2^{-2js} \lesssim2^{-2j's}\sim n^{-\frac{2s}{2s+d}} \end{aligned}$$

with $f\in B_{p,q}^{s}(H)$, Lemma 1.1, Lemma 2.2, and $t_{n}=\sqrt{\frac {\ln n}{n}}$. When $1\leq p<2$ and $s>\frac{d}{p}$, $B_{p,q}^{s}(\mathbb {R}^{d})\subseteq B_{2,\infty}^{s+d/2-d/p}(\mathbb{R}^{d})$. Then

30 $$\begin{aligned} \begin{aligned}[b] Q_{22}&\lesssim\sum_{j=j'+1}^{j_{1}} \sum_{\ell=1}^{M}\sum _{k\in \Lambda_{j}}\frac{1}{n} I_{\{|\beta_{j,k}^{\ell}|\geq\frac{\kappa t_{n}}{2}\}}\lesssim\sum _{j=j'+1}^{j_{1}}\sum_{\ell=1}^{M} \sum_{k\in \Lambda_{j}}\frac{1}{n} \biggl( \frac{\beta_{j,k}^{\ell}}{\kappa t_{n}/2} \biggr)^{p} \\ &\lesssim\sum_{j=j'+1}^{j_{1}}n^{\frac {p}{2}-1}2^{-j(s+d/2-d/p)p} \lesssim n^{\frac {p}{2}-1}2^{-j'(s+d/2-d/p)p}\sim n^{-\frac{2s}{2s+d}}. \end{aligned} \end{aligned}$$

Hence, this with (28) and (29) shows

31 $$ Q_{2}\lesssim n^{-\frac{2s}{2s+d}}. $$

Finally, one estimates $Q_{3}$. Clearly,

$$\begin{aligned} Q_{31} :=&\sum_{j=j_{0}}^{j'}\sum _{\ell=1}^{M}\sum_{k\in\Lambda _{j}} \bigl\vert \beta_{j,k}^{\ell} \bigr\vert ^{2}I_{\{|\beta_{j,k}^{\ell}|\leq 2\kappa t_{n}\}} \\ \leq&\sum_{j=j_{0}}^{j'}\sum _{\ell=1}^{M}\sum_{k\in\Lambda_{j}} |2 \kappa t_{n} |^{2} \lesssim\sum_{j=j_{0}}^{j'} \frac{\ln n}{n}2^{jd}\lesssim\frac{\ln n}{n}2^{j'd}. \end{aligned}$$

This with the choice of $2^{j'}$ shows

32 $$ Q_{31}\lesssim(\ln n)n^{-\frac{2s}{2s+d}}. $$

On the other hand, $Q_{32}:=\sum_{j=j'+1}^{j_{1}}\sum_{\ell =1}^{M}\sum_{k\in\Lambda_{j}} \vert \beta_{j,k}^{\ell} \vert ^{2}I_{\{ |\beta_{j,k}^{\ell}|\leq2\kappa t_{n}\}}$. According to the arguments of (29),

33 $$ Q_{32}\lesssim\sum_{j=j'+1}^{j_{1}} \sum_{\ell=1}^{M}\sum _{k\in\Lambda _{j}} \bigl\vert \beta_{j,k}^{\ell} \bigr\vert ^{2}\lesssim n^{-\frac{2s}{2s+d}} $$

for $p\geq2$. When $1\leq p<2$, $\vert \beta_{j,k}^{\ell} \vert ^{2}I_{\{|\beta_{j,k}^{\ell}|\leq2\kappa t_{n}\}}\leq \vert \beta _{j,k}^{\ell} \vert ^{p} \vert 2\kappa t_{n} \vert ^{2-p}$. Then similar to the arguments of (30),

34 $$\begin{aligned} Q_{32} \lesssim&\sum_{j=j'+1}^{j_{1}} \sum_{\ell=1}^{M}\sum _{k\in \Lambda_{j}} \bigl\vert \beta_{j,k}^{\ell} \bigr\vert ^{p} \vert 2\kappa t_{n} \vert ^{2-p} \\ \lesssim& \biggl(\frac{\ln n}{n} \biggr)^{\frac{2-p}{2}}\sum _{j=j'+1}^{j_{1}}2^{-j(s+d/2-d/p)p}\lesssim \biggl( \frac{\ln n}{n} \biggr)^{\frac{2-p}{2}}2^{-j'(s+d/2-d/p)p} \\ \lesssim& \biggl(\frac{\ln n}{n} \biggr)^{\frac{2-p}{2}} \biggl( \frac {1}{n} \biggr)^{\frac{(s+d/2-d/p)p}{2s+d}}\leq(\ln n)n^{-\frac{2s}{2s+d}}. \end{aligned}$$

Combining this with (33) and (32), one knows $Q_{3}\lesssim (\ln n )n^{-\frac{2s}{2s+d}}$ in both cases. This with (26), (27), and (31) shows

$$ Q\lesssim (\ln n )^{3}n^{-\frac{2s}{2s+d}}, $$

which is the desired conclusion. □
